# Histamine H_3_ receptor density is negatively correlated with neural activity related to working memory in humans

**DOI:** 10.1186/s13550-018-0406-4

**Published:** 2018-06-14

**Authors:** Takehito Ito, Yasuyuki Kimura, Chie Seki, Masanori Ichise, Keita Yokokawa, Kazunori Kawamura, Hidehiko Takahashi, Makoto Higuchi, Ming-Rong Zhang, Tetsuya Suhara, Makiko Yamada

**Affiliations:** 1 Department of Functional Brain Imaging Research, National Institute of Radiological Sciences, National Institutes for Quantum and Radiological Science and Technology, 4-9-1 Anagawa, Inage-ku, Chiba, 263-8555 Japan; 2 Department of Radiopharmaceuticals Development, National Institute of Radiological Sciences, National Institutes for Quantum and Radiological Science and Technology, 4-9-1 Anagawa, Inage-ku, Chiba, 263-8555 Japan; 3 Department of Psychiatry, Graduate School of Medicine, Kyoto University, 54 Shogoin-Kawaracho, Sakyo-ku, Kyoto, 606-8507 Japan

**Keywords:** Histamine H_3_ receptor, Working memory, PET, fMRI

## Abstract

**Background:**

The histamine H_3_ receptor is regarded as a drug target for cognitive impairments in psychiatric disorders. H_3_ receptors are expressed in neocortical areas, including the prefrontal cortex, the key region of cognitive functions such as working memory. However, the role of prefrontal H_3_ receptors in working memory has not yet been clarified. Therefore, using functional magnetic resonance imaging (fMRI) and positron emission tomography (PET) techniques, we aimed to investigate the association between the neural activity of working memory and the density of H_3_ receptors in the prefrontal cortex.

**Findings:**

Ten healthy volunteers underwent both fMRI and PET scans. The *N*-back task was used to assess the neural activities related to working memory. H_3_ receptor density was measured with the selective PET radioligand [^11^C] TASP457. The neural activity of the right dorsolateral prefrontal cortex during the performance of the *N*-back task was negatively correlated with the density of H_3_ receptors in this region.

**Conclusions:**

Higher neural activity of working memory was associated with lower H_3_ receptor density in the right dorsolateral prefrontal cortex. This finding elucidates the role of H_3_ receptors in working memory and indicates the potential of H_3_ receptors as a therapeutic target for the cognitive impairments associated with neuropsychiatric disorders.

## Findings

### Background

Working memory, the ability to retain information for a short period of time [1], is regarded as a core cognitive function that underpins a wide range of complex behaviours such as problem solving, decision-making and reasoning. A substantial number of neuroimaging studies using fMRI have shown that the dorsolateral prefrontal cortex (DLPFC) is the key cortical region involved in working memory [2]. Moreover, several neurotransmitters are known to be involved in this process.

Among the multiple neurotransmitter systems, components of histaminergic neurotransmission, particularly H_3_ receptors, are known to modulate working memory in animals [3]. The H_3_ receptor is a presynaptic receptor that regulates the release of histamine (as an autoreceptor) as well as other neurotransmitters such as dopamine, norepinephrine and acetylcholine (as a heteroreceptor), which are involved in cognitive function [3]. More specifically, increased release of histamine via H_3_ receptor antagonists has been shown to improve working memory in rats under various maze tasks and the delayed match-to-sample test [3]. Thus, extensive preclinical studies have assessed the role of H_3_ receptors in working memory. However, very few such studies have been conducted in humans, and the published clinical studies of H_3_ receptor antagonist/inverse agonists have shown the mixed results of cognitive improvements in neuropsychiatric diseases ([4, 5] for review). For example, some studies reported the positive effects of H_3_ receptor drugs in episodic memory in Alzheimer’s disease, while others found no beneficial effects examined by various drugs in different diseases [5]. These unclear therapeutic effects may come from the complex biology and pharmacology of H_3_ receptor, such as the heterogeneity of isoforms and the different profile of drug activity (full agonists, partial agonists, neutral antagonists, inverse agonists and protean ligands) [5].

Nevertheless, H_3_ receptors are highly expressed in the human cerebral cortex and basal ganglia, as revealed by autoradiographic studies of post-mortem brain tissue and by recent PET studies using radioligands targeting H_3_ receptors [3, 6]. Furthermore, a post-mortem brain sample study revealed that the prefrontal cortex of schizophrenia patients with cognitive impairments showed increased H_3_ receptor binding [7].

Thus, because the prefrontal cortex is the key region for working memory and H_3_ receptors are highly expressed in this region, the present study aimed to clarify whether brain activity related to working memory was associated with H_3_ receptor density in the prefrontal cortex. To accomplish this, we used fMRI as well as PET with the radioligand [^11^C] TASP457, which has high affinity and selectivity for H_3_ receptors [6, 8].

## Methods

### Participants

Ten right-handed (self-reported) healthy male volunteers (mean age ± standard deviation, 25 ± 4 years) participated in the study. All participants had no history of neurological and psychiatric disorders and were not taking any medications. The subjects underwent an fMRI with *N*-back task followed by a PET scan at rest, with the mean interval of 18.8 ± 20.6 days (mean ± standard deviation).

### Radioligand preparation

The [^11^C] TASP457 precursor and standard used in this study were provided by Taisho Pharmaceutical Co., Ltd. [^11^C] TASP457 was radiosynthesised by O-alkylation of the 2-pirydone-containing precursor (desmethyl TASP457) with [^11^C] methyl triflate [4]. At a time of administration, the radiochemical purity of [^11^C] TASP457 was > 95%, and its specific activity was > 37 GBq/μmol.

### PET procedures

After intravenous injection of [^11^C] TASP457 (390 ± 6 MBq with molar activity of 92 ± 32 GBq/μmol), we acquired three-dimensional dynamic images with a PET camera (Eminence SET-3000GCT/X, Shimadzu, Kyoto, Japan) for 120 min in 39 frames of increasing duration (from 10 s to 5 min). All PET images were reconstructed using the filtered back-projection method (Gaussian filter, kernel 5 mm; the reconstructed in-plane resolution was 7.5 mm in full width at half maximum (FWHM); voxel size 2 × 2 × 2.6 mm) corrected for attenuation, randoms, scatter and head motion. Plasma input functions were measured with arterial blood sampling and metabolite analyses using a radio high-performance liquid chromatography, as described previously [6].

### fMRI procedures

We designed a modified version of the *N*-back task used in a previous study [2]. Participants responded to the numeric characters of previously seen stimuli according to three conditions, 0-, 1- and 2-backs.

A Siemens Verio 3 T MRI system (Siemens, Erlangen, Germany) was used to obtain T2*-weighted echo-planar imaging (repetition time [TR] = 2000 ms, echo time [TE] = 25 ms, slice number = 33 (interleaved), slice thickness = 3.8 mm, matrixes = 64 × 64, 345 volumes) with bold oxygen level-dependent (BOLD) contrasts and structural T1 image (TR = 2300 ms, TE = 1.95 ms, slice number = 176, slice thickness = 1 mm, matrixes = 256 × 256).

Preprocessing analysis with SPM8 software (Wellcome Department of Cognitive Neurology, London, UK) included slice time correction, realignment, DARTEL normalisation and smoothing with a 6-mm FWHM Gaussian kernel. First-level analysis modelled the task as a block design, with working memory load as a linear regressor (0-back = − 1, 1-back = 0, 2-back = 1). Six realignment parameters and two derivatives were used as covariates. Artefacts in fMRI time series data were detected and corrected using robust weighted least squares [9]. A mask image of the prefrontal cortex (including Brodmann areas 8, 9, 10, 11, 12, 13, 14, 24, 25, 32, 44, 45, 46 and 47) was created using WFU PickAtlas 3.0.5 software (Wake Forest University, Winston-Salem, NC). Group-level random effect analysis was performed to identify the activity corresponding to increased working memory load within the prefrontal cortex, using a threshold of *P* < 0.001 (uncorrected) with a minimum cluster size of 20 voxels [10]. Age was included as a nuisance covariate.

### PET and fMRI analyses

The contrast coefficients (*ß* value) were extracted from the cluster images of increased working memory load. Time–activity curves were generated using data extracted from the PET images by applying the spherical (radius 4 mm) regions of interest images centred on the peak coordinates of each cluster. Total distribution volume (*V*_T_), which reflects the H_3_ receptor density in the brain, was calculated using Ichise’s multilinear analysis (MA1) [11] with the time–activity curves for the initial 60 min and plasma input functions according to the previous quantitative analysis of [^11^C] TASP457–PET data [6]. The correlation analyses between MRI (*ß* value) and PET (*V*_T_) data were conducted with IBM SPSS Statistics, version 23 (IBM Corp., Armonk, NY). To compensate for the small sample size, we used a resampling procedure based on 5000 bootstrapped samples, using bias-correlated and accelerated (BCa) 95% confidence intervals (CIs) to estimate Pearson’s correlation coefficient for the neural activity and *V*_T_.

## Results

### Behavioural data

There were no significant differences in accuracy across the three *N*-back tasks (0-back, 98.0 ± 0.7%; 1-back, 98.1 ± 0.8; 2-back, 97.1 ± 1.1; Friedman test *P* = 0.66, *χ*^2^ = 0.84, df = 2). The performances of 1- and 2-backs showed the ceiling effects as they were not significantly different from the maximum accuracy of 100% (Wilcoxon signed-rank tests both *P*s > 0.05, both *W*s = − 15); thus, the behavioural data were not used for further analyses to avoid spurious estimates.

### Imaging data

**Fig. 1:**
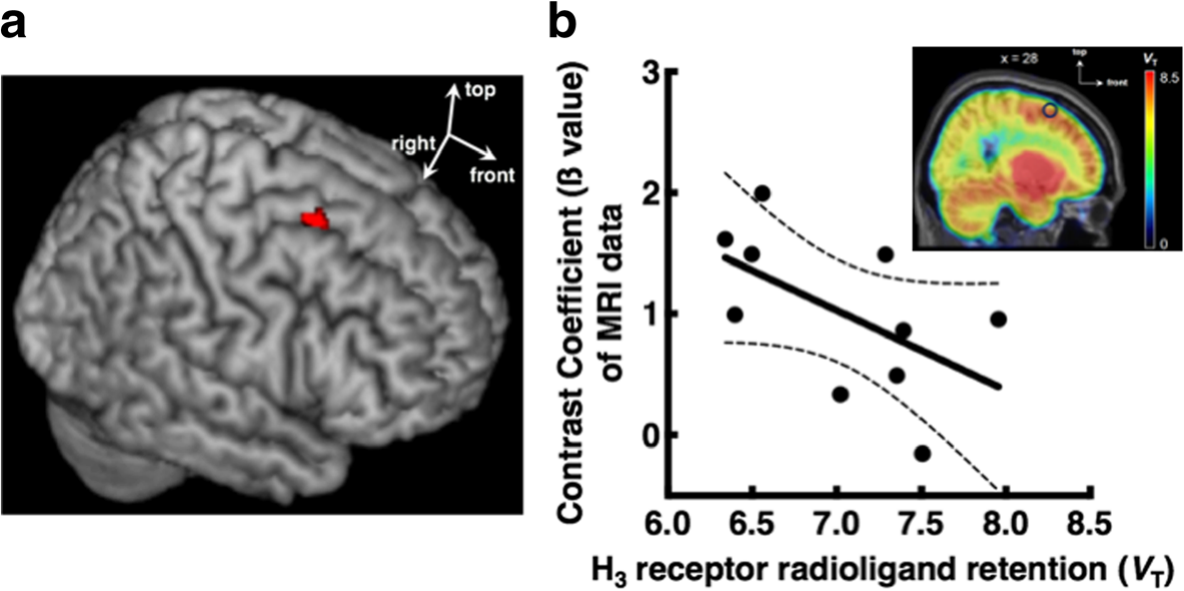
**a** Group map showing the activity in the right middle frontal gyrus (red, *x*, *y*, *z* = 28, 10, 60) in response to increased working memory load. **b** The activity in the right middle frontal gyrus was negatively correlated with the H_3_ receptor radioligand retention (*V*_T_), which reflects the H_3_ receptor density. The dashed lines indicate 95% confidence intervals. The PET image on the graph indicates the distribution of the H_3_ receptors in the brain. The sagittal image was obtained by averaging parametric images of the 10 subjects. The parametric images were calculated using Ichise’s multilinear analysis to estimate total distribution volume for each voxel and spatially normalised thereafter. The circle indicates the location of the activated region detected in fMRI during performing working memory task in the right middle frontal gyrus shown in **a**

Three clusters, in which neural activities assessed by fMRI were associated with increased working memory load, were detected in the bilateral DLPFC (the left middle frontal gyrus, left superior frontal gyrus and right middle frontal gyrus; Table 1). Their *V*_T_ values (mL/cm^3^), estimated from PET data, were 7.4 ± 0.6 for the left middle frontal gyrus, 7.2 ± 0.9 for the left superior frontal gyrus and 7.0 ± 0.6 for the right middle frontal gyrus. The *ß* value of the right middle frontal gyrus (Fig. 1a) was negatively correlated with the *V*_T_ value of this region (Spearman *r* = − 0.65, *P* = 0.043, 95% BCa CI = (− 0.91, − 0.05), bias = 0.063, standard error = 0.21, Fig. 1b). No significant correlations were found in the other two clusters.

**Table 1:** Neural activity as a function of increased working memory load

Region	Side	BA	Cluster size	*Z* value	*x*	*y*	*z*
Middle frontal gyrus	Left	9	83	3.91	− 28	32	34
Superior frontal gyrus	Left	8	45	3.53	− 26	8	60
Middle frontal gyrus	Right	8	21	3.33	28	10	60

(*x*, *y*, *z*) corresponds to Montreal Neurological Institute (MNI) coordinates

*BA* Brodmann area

## Discussion

The present study revealed that higher activity in the right DLPFC due to increased working memory load, which was consistent with the findings of previous studies [1, 2], was associated with lower density of H_3_ receptors. Preclinical studies demonstrated that H_3_ receptor blockade with H_3_ antagonists increased the release of neurotransmitters such as acetylcholine and dopamine, resulting in enhanced cognition, whereas H_3_ agonists impaired cognition [3]. Enhanced cognition has also been reported in H_3_ receptor knockout rodents [3]. Thus, the individual variability of H_3_ receptor density may reflect the ability of neurotransmitter release. Taken together with our findings, these results indicate that inhibition of H_3_ receptors, which increases the release of histamine and other neurotransmitters, plays a role in working memory activation in the right DLPFC.

Although very few studies have investigated the role of H_3_ receptors in working memory in humans, one fMRI study revealed that an increase in histamine neurotransmission induced by betahistine (H_3_ antagonist/H_1_ agonist) moderately increased right DLPFC activity during *N*-back task performance [12]. This finding also supports the role of the H_3_ receptor inhibition in DLPFC activation, consistent with our study results.

One of the key limitations of this study is its small sample size, which decreased its statistical power. Moreover, we were unable to establish a causal relationship between H_3_ receptor density and working memory activity in the DLPFC or to examine the association between H_3_ receptors and working memory performance. Further studies with a larger sample size and the use of H_3_ agonists/antagonists are required to establish the causal relationship between H_3_ receptors and working memory activity and its performance.

## Conclusion

In conclusion, the present study showed for the first time that H_3_ receptor density was associated with working memory activity in the right DLPFC in humans, revealing a histaminergic mechanism underlying working memory. This finding supports the potential of histaminergic modulators, especially those that affect H_3_ receptors, for treating cognitive impairments in neuropsychiatric disorders.
